# Whole exome sequencing detects homozygosity for ABCA4 p.Arg602Trp missense mutation in a pediatric patient with rapidly progressive retinal dystrophy

**DOI:** 10.1186/1471-2350-15-11

**Published:** 2014-01-20

**Authors:** Maria Carolina Ortube, Samuel P Strom, Stanley F Nelson, Steven Nusinowitz, Ariadna Martinez, Michael B Gorin

**Affiliations:** 1 Department of Ophthalmology, Jules Stein Eye Institute, David Geffen School of Medicine at University of California, Los Angeles, CA DS-2-545, USA; 2 Department of Pathology and Laboratory Medicine, David Geffen School of Medicine at University of California, Los Angeles, USA; 3 Department of Human Genetics, David Geffen School of Medicine at University of California, Los Angeles, USA

**Keywords:** ABCA4 retinopathy, Pediatric, Homozygosity, Consanguinity

## Abstract

**Background:**

A pediatric patient presented with rapidly progressive vision loss, nyctalopia and retinal dystrophy. This is the first report of homozygosity for the p.Arg602Trp mutation in the *ABCA4* gene. The child became legally blind within a period of 2 years.

**Case presentation:**

An eight year-old Hispanic female presented with bilateral decreased vision following a febrile gastrointestinal illness with nausea and vomiting. Extensive workup involved pediatric infectious disease and rheumatology consultations.

Initial visual acuity was 20/60 at distance and 20/30 at near in both eyes. Rapidly progressive vision loss occurred during a 2-year period resulting in visual acuities of 20/200 at distance in both eyes. Fundus exam disclosed attenuated vessels and multiple subretinal blister-like elevations. Optical coherence tomography showed far more lesions than were clinically evident with different levels of elevation. Autofluorescence imagery showed dramatic and widespread geographic areas of atrophy. The deposits that appeared drusen-like on clinical exam were hyperfluorescent, consistent with lipofuscin deposits containing A2e (N-retinylidene-N-retinylethanolamine) indicative of RPE cell dysfunction. Electroretinography was consistent with cone dystrophy, with relative preservation of rod function. Blood analysis and rheumatology evaluation found no evidence of a diffuse post-infectious/inflammatory process. The unique and rapid progression of her subretinal blister-like lesions was documented by fluorescein angiography, optical coherence tomography, autofluorescence imagery, and fundus photography. Family pedigree history disclosed consanguinity, her parents being first cousins. DNA analysis by whole exomic sequencing revealed homozygosity of p.Arg602Trp in the ABCA4 gene.

**Conclusion:**

The pediatric patient presented with a striking clinical appearance and dramatic rate of progression that was clinically more characteristic of an infectious or inflammatory process. This case expands the diverse range of phenotypes attributed to *ABCA4* mutations and further supports the role of whole exome sequencing as a powerful new tool available to aid clinicians in establishing diagnosis for challenging cases.

## Background

*ABCA4* related retinopathies include an interesting diversity of common and unusual retinal phenotypes [1]. Mutations in the *ABCA4* gene have been linked to several conditions such as autosomal recessive retinitis pigmentosa (arRP) [2], autosomal recessive Stargardt disease (arSTGD), autosomal recessive cone-rod dystrophy (arCRD) [3], bull’s eye maculopathy [4], and age-related macular degeneration [5,6].

Stargardt disease is the most common hereditary macular dystrophy, affecting children with a prevalence of 1:10.000, as described by September et al. [7].

In this report we present a case of early onset and dramatic vision loss progression in a child who underwent multiple ophthalmological and systemic procedures in order to rule out neoplastic, inflammatory or infectious etiologies. After 2 years of ophthalmic follow-up we performed exomic sequencing and identified a likely pathogenic homozygous variant in *ABCA4* (p.Arg602Trp)*.*

This variant has been reported previously in *homozygous* form but, except for age of onset, the phenotype associated with this variant is not well described [7,8].

Here we provide a comprehensive phenotypic characterization of a young patient *homozygous* for this highly pathogenic p.Arg602Trp variant.

## Case presentation

### Materials and methods

The study was carried out with approval of UCLA Institutional Review Board (IRB) and the study was conducted in accordance with regulations of the Health Insurance Portability and Accountability Act of 1996 (HIPAA).

The proband and her parents enrolled in the Genetics research study and signed a consent form. The UCLA HIPAA consent form signed by her parents specifies the use of the information for publications and research presentations.

Retrospective case report: An eight year-old Hispanic female presented with bilateral decreased vision that was detected one month after an acute febrile gastrointestinal illness. Initial extensive workup involved pediatric infectology, rheumatology and ophthalmology consultations.

### Ophthalmic diagnostics

Fundus photography and fluorescein angiography were obtained with ultra wide-field Scanning laser ophthalmoscopy (SLO) (Optos P200C).

Retinal structure was documented by spectral domain optical coherence tomography (sdOCT) using an extended 25-degree macular cube with 25 slices (*Spectralis*, Heidelberg Engineering, Heidelberg, Germany).

Full-field ERGs were recorded in standardized fashion [9] from both eyes after pupil dilation as previously described [10,11].

Autofluorescence & infrared imagery was obtained with HRAII (30 & 55 degree field of views) (Heidelberg Engineering) as previously described [12].

Humphrey visual field testing was performed with 24–2 full threshold or SITA [Swedish Interactive Thresholding Algorithm] standard program (Carl Zeiss Meditec).

Central color vision was assessed with the Farnsworth saturated D-15 and the Ishihara Pseudo-Isochromatic color plates presented under appropriate lighting conditions.

### Genetic testing

The patient underwent genetic testing for *VMD2* gene in order to rule out Best disease.

### Whole exome sequencing data acquisition

Standardized protocols were used to generate whole exome sequencing (WES) data, as follows. Randomly fragmented genomic DNA libraries were created following standard protocols for high-throughput paired-end sequencing on the HiSeq2000 instrument (*Illumina Inc*., San Diego, CA). The Agilent SureSelect 50Mb capture kit was used to enrich the libraries for known coding loci in the human genome (Agilent Technologies; Santa Clara, CA). This kit has been shown to effectively capture >90% of these loci when an adequate average read depth is reached [13].

### Whole Exome Sequencing (WES) data analysis

Nucleotide base calling and quality score assessment was performed using instrument-specific Real Time Analysis (RTA) software provided by Illumina. A combination of commercially or academically available tools and custom scripts were used to analyze the raw DNA sequence reads. Alignment to the human genome (hg19; NCBI build 37; Feb. 2009) was performed using Novoalign V2.07.15b (Novocraft Technologies; Selangor, Malaysia). Merging, sorting, and other manipulation of aligned data was performed using SAMTools [14]. PCR clonal duplicate removal was performed using Picard -tools-1.42 (freeware) [http://picard.sourceforge.net]. Quality score recalibration, genotyping, variant filtration, and coverage depth analysis were performed using the Genome Analysis Toolkit (GATK v1.1) [15].

Variant consequence analysis was performed using SeattleSeq v7.0 (University of Washington) [16] which incorporates many databases including: “NCBI full genes”, dbSNP131, and the 1000 Genomes Project.

The following cutoffs were used to identify high-priority candidate variants: homozygous, Quality Score ≥ 100, coverage depth ≥ 20, not overlapping a segmental duplication (USCS Genome Browser Assembly GRCh37), minor allele frequency < 0.01 (dbSNP135). A custom PERL script was used to search for runs of homozygosity. A run of homozygosity was defined here as any interval larger than two million base pairs (Mb) in which greater than 95% of all non-reference single nucleotide variants (SNVs) were homozygous. Familial cosegregation analysis was used.

## Results

The vision loss was detected one month after an acute gastrointestinal illness with high fever, headaches, nausea and vomiting that required emergency room consultation. The child developed acute symptoms after ingesting poultry imported from El Salvador, creating the initial suspicion of gastrointestinal infection. Bilateral uncorrected visual acuity was 20/50 at distance and 20/25 at near. Visual fields showed scattered scotomas in both eyes with central vision loss in an ill-defined pattern consistent with retinal dysfunction. A review of prior fundus exam images showed focal heterogeneous pigmentary changes in the superotemporal region in both eyes. Electroretinography testing performed at 4 months after initial consultation showed severely decreased amplitude and delayed implicit time for cone function, with relative preservation of rod function.

Farnsworth D-15 color testing was abnormal for both eyes, the errors were distributed around the spectrum with a suggestion of a tritan axis. Only two of the 24 Ishihara color plates were identified correctly with the right eye and only three with the left eye. Sagittal and axial contrast brain magnetic resonance imaging was within normal limits.

The child was referred to UCLA for a third opinion consultation 6 months after the onset of vision loss and nyctalopia. Family history was significant for first cousin consanguinity between the child’s parents, but there was no family history of blindness. Bilateral distance visual acuity was 20/60, improving to 20/50 by pinhole. Bilateral near visual acuity was 20/30.

Cycloplegic retinoscopy detected very mild hyperopia and astigmatism in both eyes (OD: + 1.00 sphere + 1.00 cylinder axis 110 degrees; OS: + 0.75 sphere + 0.50 cylinder axis 60 degrees). There was no afferent pupillary defect. Ocular versions and saccadic movements were full and the child was orthotropic at distance and near. Stereopsis was 3000 arc seconds (Titmus test).

Anterior segment exam was unremarkable. The vitreous was clear without cells.

Fundus exam disclosed attenuated vessels in all quadrants and multiple scattered cysts or “blister-like” subretinal elevations, more evident in the posterior pole, with extensive geographic areas of pigment epithelial deposits. Cup to disc ratio was 0.6 in both eyes. The foveal reflex was relatively normal in the right eye and decreased in the left eye.

**Figure 1:**
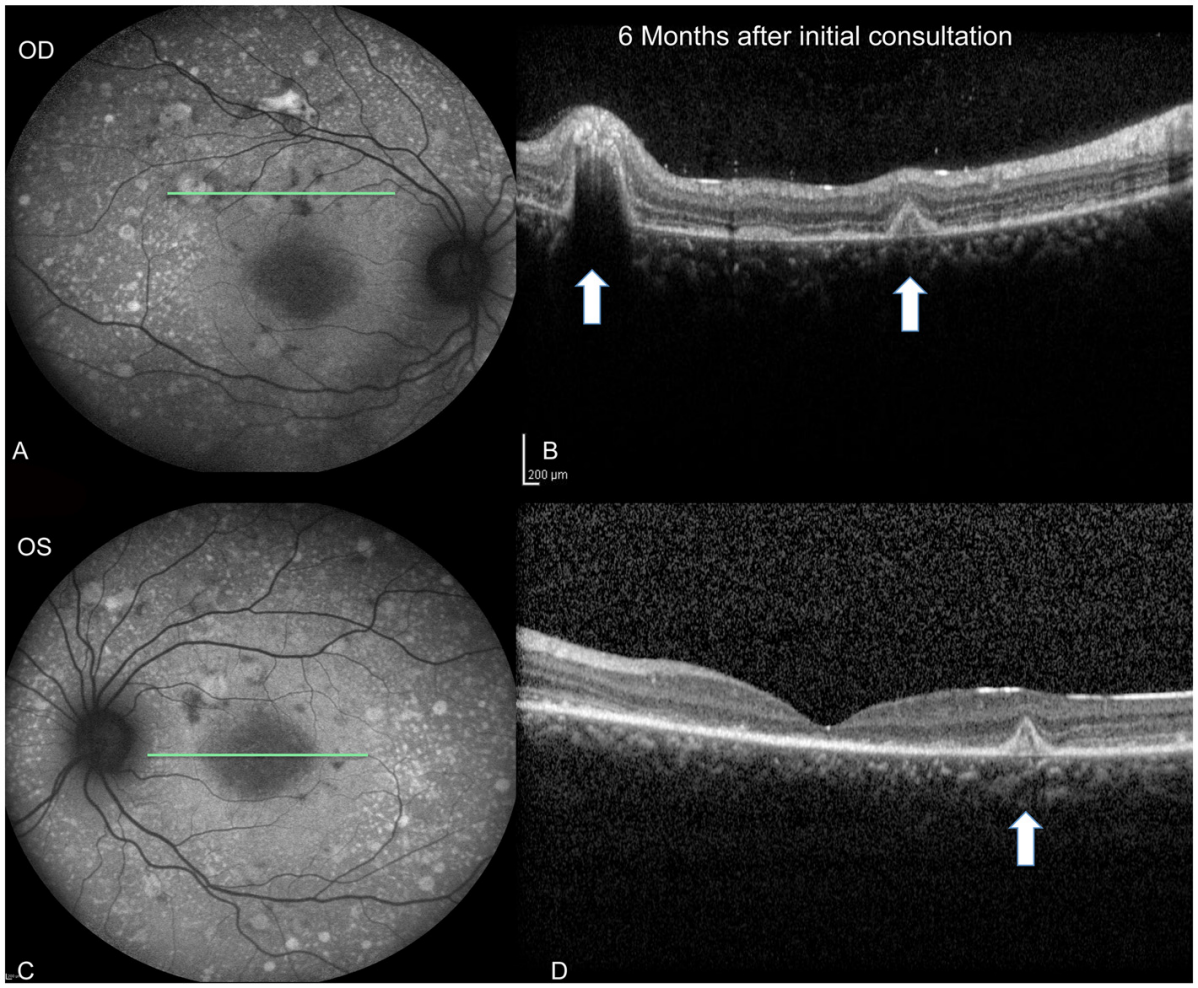
**55 degrees autofluorescence (A: Right eye & C: Left eye) and optical coherence tomography (B: Right eye & D: Left eye) performed 6 months after initial consultation.** The OCT scan line location is shown as a green line in the autofluorescence images **(A & C)**. The deposits that appeared drusen-like on clinical exam were hyperfluorescent on autofluorescence imagery **(A & C)**, indicative of RPE cell dysfunction. The OCTs revealed numerous subretinal lesions with different levels of elevation throughout the posterior pole. White arrows point to three “blister” like lesions, as seen by OCT. (**B**, right eye &**D**, left eye).

55 degrees autofluorescence imagery performed at 6 months after initial consultation (see Figure 1A: Right eye/C: Left eye) showed dramatic and widespread regions of both hyperfluorescence and hypofluorescence. The deposits that appeared drusen-like on clinical exam were hyperfluorescent, consistent with lipofuscin deposits containing A2E (N-retinylidene-N-retinylethanolamine) indicative of RPE cell dysfunction [12].

The OCT images revealed numerous subretinal “Blister-like” lesions with different levels of elevation throughout the posterior pole (see Figure 1B, right eye and D: left eye).

Rheumatology evaluation found no evidence of a diffuse post-infectious/inflammatory process. Blood testing was negative for bacteria, parasites or virus except for cytomegalovirus (Positive IgG, negative IgM).

The patient experienced rapidly progressive vision loss during a 2-year period with visual acuities reduced to 20/200 at distance in both eyes from 20/50 at first testing.

The unique and rapid centrifugal progression of her subretinal blister-like lesions and fibrotic scarring was documented by fundus photography, OCT, autofluorescence and fluorescein angiography.

**Figure 2:**
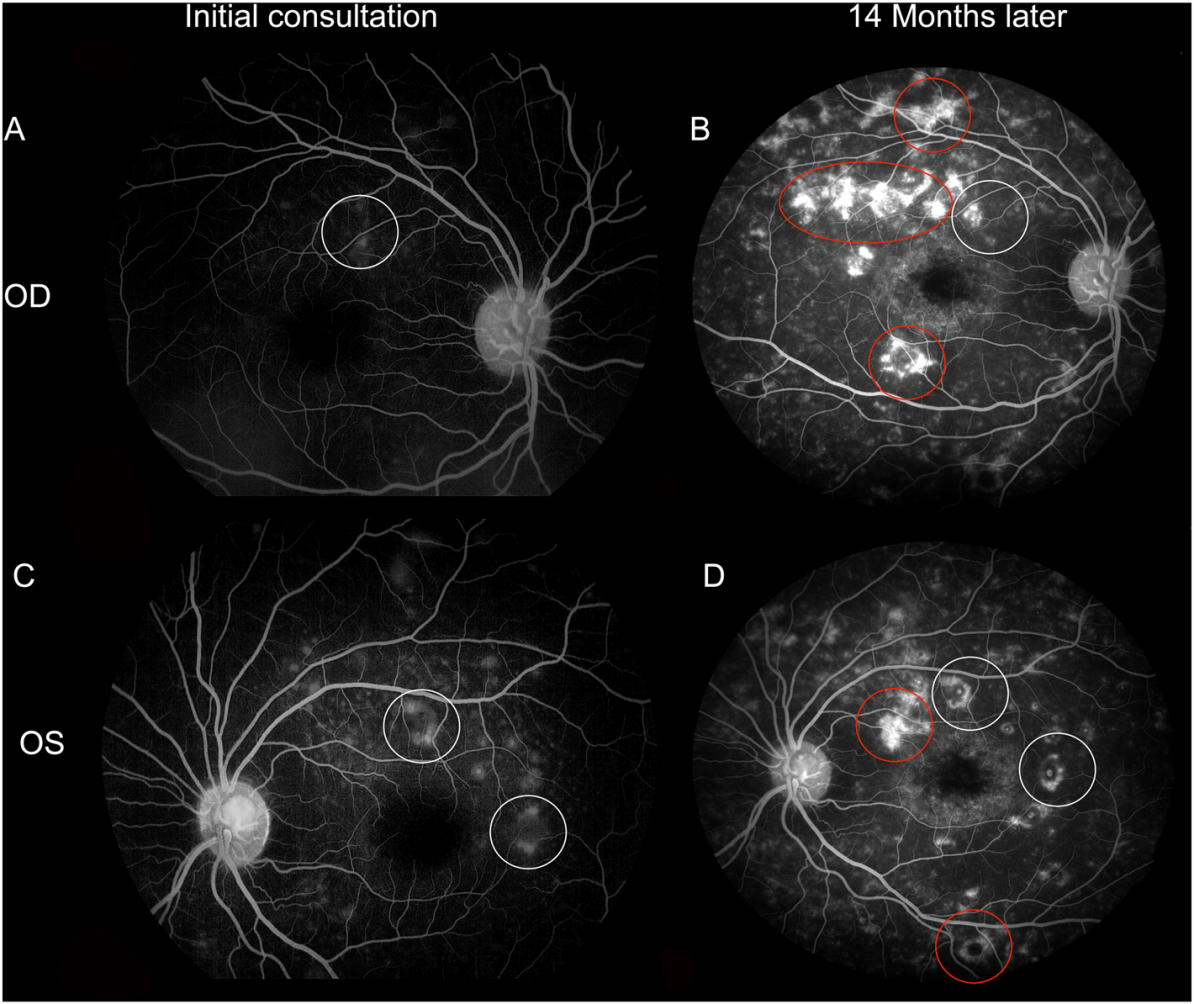
**Fluorescein angiography at initial ophthalmic consultation (Late phase, A: Right eye & C: Left eye).** The left eye was initially more affected than the right eye, showing multiple ill-defined hyperfluorescent lesions (as described within the boundaries of white circles), with a dark choroid impression. Fluorescein angiography was repeated at UCLA 14 months after initial consultation (Late phase, **B**: Right eye and **D**: Left eye). The angiogram revealed more lesions than were apparent by ophthalmoscopy. There was a marked progression in the number and size of the hyperfluorescent subretinal lesions and fibrotic scarring in the posterior pole and peripheral retina (As shown within the boundaries of red circles & ellipses to describe new lesions; white circles describe the old lesions for comparison). There was no extravasation of the dye in the late phase.

Figure 2 describes the fluorescein angiography comparison at initial consultation (A, right eye and C, left eye) and 14 months later (B, right eye and D, left eye). Initially, the subretinal lesions appeared hyperfluorescent on fluorescein angiography test with a dark choroid impression. There was a marked progression in the number and size of hyperfluorescent lesions, but there was no extravasation in the late phase.

**Figure 3:**
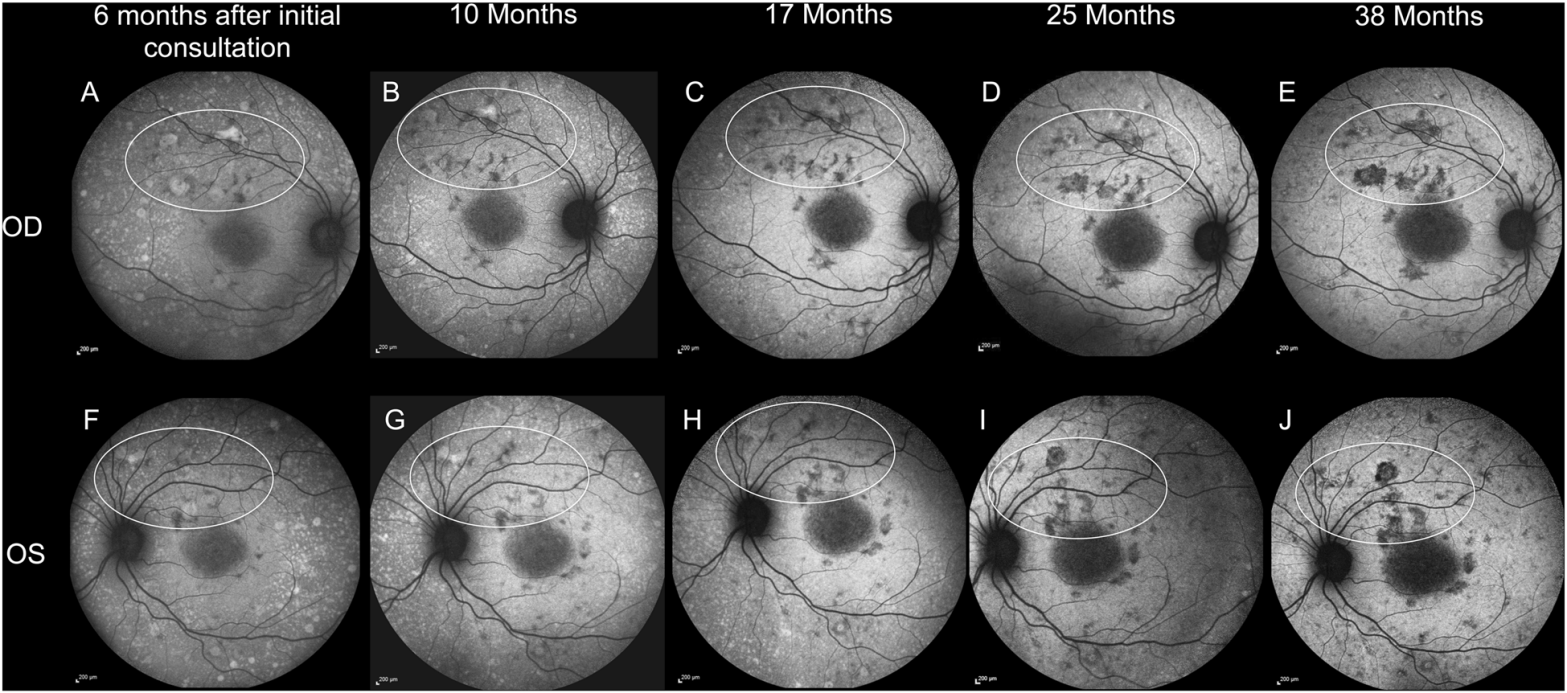
**(55 degrees version).** 55 degrees Autofluorescence **(AF)** imagery for the right eye (Top panel) and the left eye (Lower panel) at 6 **(A, F)**, 10 **(B, G)**, 17 **(C, H)**, 25 **(D, I)** and 38 months **(E, J)** after initial consultation. Note the dramatic change of size and spatial distribution of hyperfluorescent and hypofluorescent lesions (as shown within the boundaries of ellipses). The reduced AF areas are presumed to identify regions in transition to geographic atrophy onset. Note that the bilateral hyperfluorescent lesions temporal to the fovea at 6 months after initial consultation **(A & F)** are much less apparent by 38 months **(E & J)**.

Figure 3 shows the 55 –degrees autofluorescence (AF) imagery for the right eye (Top panel) and the left eye (Lower panel) at 6, 10, 17, 25 and 38 months after the initial consultation. Note the dramatic change in size and spatial distribution of hyperfluorescent and hypofluorescent lesions (as shown within the boundaries of ellipses and circles). The reduced AF areas are presumed to identify regions in transition to areas of GA onset. Interestingly, there is a bilateral region of hyperfluorescent “Blister-like” lesions temporal to the fovea at 6 months after initial consultation that slowly became less apparent by 38 months.

**Figure 4:**
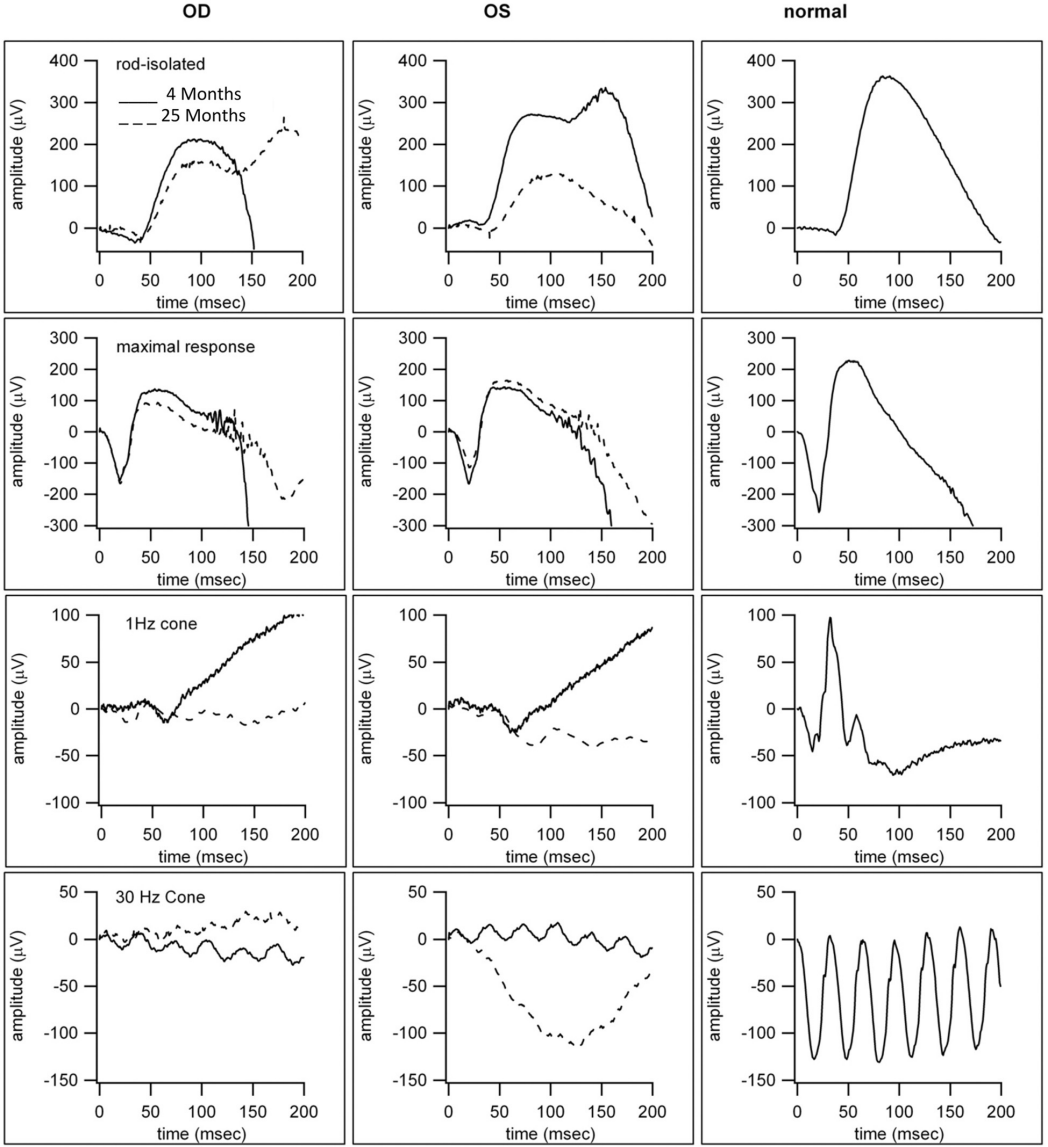
**Shows the normal electroretinogram (ERG) responses for a child at this age and the ERG responses obtained at 4 months and 25 months after initial consultation.** The ERG is consistent with a modest decrease in both rod and cone mediated retinal function over the course of 21 months.

Figure 4 shows the results of ERG testing 4 months after the initial consultation and 21-months later. A representative age-matched normal ERG is shown in the right column for comparison.

The ERGs recorded on both visits demonstrated severely truncated and delayed cone responses, worse for the right eye, with relatively preserved, although abnormal rod function. In contrast to the AF imaging that suggested rapid progression of disease (as described above), a significant progression of ERG responses was not apparent.

Near IR AF performed at 38 months after initial consultation showed far more lesions that were clinically evident by standard AF imagery.

At that time, the visual acuity remained stable at 20/200 and the child was using a low vision device to read.

### Genetic testing

*VMD2* testing for vitelliform macular dystrophy (Best disease) was negative. Whole exome sequencing identified a homozygous *ABCA4* missense variant (p.Arg602Trp) that has been identified as a Stargardt Disease mutation [7,8]. Familial cosegregation analysis was used, with both parents being heterozygous carriers.

Homozygosity mapping identified 23 blocks (> 2 Mb) of autozygosity mapping to ten different chromosomes. Approximately 208 Mb or ~7% of the genome was located within such a block in this individual. One of the largest blocks mapped to the proximal arm of chromosome 1 (1p31.1-p22.1; 74.2 Mb-94.6; ~20.5 Mb in size) and included *ABCA4* at its distal end, among many other genes. This degree of autozygosity strongly confirmed parental consanguinity at the level of first cousins or closer, consistent with the family self-report.

Whole exomic sequencing (WES) identified a total of 14 homozygous rare coding variants, including p.Arg602Trp in *ABCA4* (Additional file 1)*.* Targeted loci were covered on average by 105 independent sequence reads. This level of coverage exceeds minimum recommendations for recessive disease allele discovery [13].

Variants were not filtered for zygosity. All variants observed within known retinal disease genes were subject to interpretation. No other variants or sets of variants were identified which were consistent with a genetic disorder (e.g. compound heterozygous or homozygous variants in recessive genes or likely causal heterozygous variants in dominant disease genes). The total number of single-nucleotide variants observed within targeted protein-coding loci was 20,478 (Qscore ≥ Q100). Over 95% of these variants mapped to common polymorphisms in dbSNP131. A total of 592 small insertions or deletions (indels) were also observed. These values are typical for WES experiments. Compound heterozygous cases that included the p.Arg602Trp mutation of the *ABCA4* gene have been described in early onset, autosomal recessive retinitis pigmentosa families [7,8]. This variant has been previously reported in a homozygous state. Given the patient’s unique ocular phenotype, the age of onset and the phenotypic variability observed in *ABCA4-*related disease [4], we conclude that this homozygous variant is likely disease-causing and rapidly progressive.

## Conclusion

In children, it is important to consider genetic etiologies in the presence of unusual phenotypes with sudden onset vision loss. In our clinical report, the combination of ocular history, fundus imaging and electroretinography led to a diagnosis of a rapidly progressive retinopathy in a young patient with a severe *ABCA4* homozygous variant.

In a case of rapidly progressive retinal degeneration, Batten disease (neuronal ceroid lipofuscinosis) should be suspected. Fortunately, the child did not present with seizures or neurologic impairment, so no genetic testing was required to rule out this diagnosis.

The presence of fibrotic scarring in the paramacular area [17], the dark choroid, the autofluorescence phenotype and the ERG results are consistent with an *ABCA4* related retinopathy. The centrifugal expansion of fundus autofluorescence patterns in Stargardt disease has been described previously by Cukras et al. [12,18].

All individuals contain a substantial number of potential disease-causing variants in their DNA and it is not surprising that an offspring of a consanguineous mating would be homozygous for several potentially deleterious recessive alleles. In addition to the aforementioned variants in *ABCA4*, this patient also harbored 14 homozygous, rare variants that alter the amino acid sequences of the encoded proteins (Additional file 1). The extent to which these and other heterozygous genetic variants contribute to the systemic and ophthalmic clinical well being of this patient is not discernable at this time.

The value of exome sequencing is crucial in cases where the phenotype is not suggestive of a particular candidate gene or set of genes, and this approach allows one to reasonably address multiple genetic etiologies. However it is also important to emphasize that, given the complexity of the data provided by exome sequencing, a careful pedigree, clinical ascertainment of other family members and the testing of DNA from key family members is essential for interpreting that data.

## Competing interests

The authors declare that they have no competing interests.

## Authors’ contributions

MCO examined the proband, conceived the study, enrolled the proband and parents in the study, drafted the manuscript, created the study figures. Lecturer at the Second World Congress of Pediatric Ophthalmology and Strabismus, Medical retina symposium. September 2012, Milan, Italy. SPS carried out the molecular genetic studies, analyzed the genetic results & helped to draft the manuscript. SFN carried out the molecular genetic studies & helped to draft the manuscript. SN carried out the electroretinography studies, worked on the figures & helped to draft the manuscript. AM participated in the design and coordination of the study, provided genetic counseling to the family, helped to draft the manuscript and to analyze the genetic information. MBG conceived the study, examined the proband, and helped to draft the manuscript. All authors read and approved the final manuscript.

## Pre-publication history

The pre-publication history for this paper can be accessed here:

http://www.biomedcentral.com/1471-2350/15/11/prepub

## Supplementary Material

Additional file 1 **Information for the 14 homozygous variants identified in subject meeting inclusion criteria: Quality Score ≥100, coverage depth ≥ 20, no overlap with segmental duplications (UCSC Genome Browser), Minor Allele Frequency <0.01 (dbSNP135).** The putative causal variant in ABCA4 is highlighted in bold. No other variants are clinically relevant to the patient’s presentation. Abbreviations: Chr, chromosome; Ref, human genome reference nucleotide; Alt, alternate allele nucleotide; OMIM, Online Mendelian Inheritance in Man database; STGD, Stargardt Disease; OMD, Occult Macular Dystrophy; US1D, Usher Syndrome Type 1D. Click here for file

## References

[B1] Duno M Schwartz M Larsen PL Rosenberg T Phenotypic and genetic spectrum of Danish patients with ABCA4-related retinopathy Ophthalmic Genet 2012 15 4 225 231 10.3109/13816810.2011.643441 22229821

[B2] Cremers FP van de Pol DJ van Driel M den Hollander AI van Haren FJ Knoers NV Tijmes N Bergen AA Rohrschneider K Blankenagel A Pinckers AJ Deutman AF Hoyng CB Autosomal recessive retinitis pigmentosa and cone-rod dystrophy caused by splice site mutations in the Stargardt’s disease gene ABCR Hum Mol Genet 1998 15 3 355 362 10.1093/hmg/7.3.355 9466990

[B3] Klevering BJ Deutman AF Maugeri A Cremers FP Hoyng CB The spectrum of retinal phenotypes caused by mutations in the ABCA4 gene Graefes Arch Clin Exp Ophthalmol 2005 15 2 90 100 10.1007/s00417-004-1079-4 15614537

[B4] Michaelides M Chen LL Brantley MA Jr Andorf JL Isaak EM Jenkins SA Holder GE Bird AC Stone EM Webster AR ABCA4 mutations and discordant ABCA4 alleles in patients and siblings with bull’s-eye maculopathy Br J Ophthalmol 2007 15 12 1650 1655 10.1136/bjo.2007.118356 18024811 PMC2095527

[B5] Allikmets R Shroyer NF Singh N Seddon JM Lewis RA Bernstein PS Peiffer A Zabriskie NA Li Y Hutchinson A Dean M Lupski JR Leppert M Mutation of the Stargardt disease gene (ABCR) in age-related macular degeneration Science 1997 15 5333 1805 1807 10.1126/science.277.5333.1805 9295268

[B6] Fritsche LG Fleckenstein M Fiebig BS Schmitz-Valckenberg S Bindewald-Wittich A Keilhauer CN Renner AB Mackensen F Mößner A Pauleikhoff D Adrion C Mansmann U Scholl HP Holz FG Weber BH A subgroup of age-related macular degeneration is associated with mono-allelic sequence variants in the ABCA4 gene Invest Ophthalmol Vis Sci 2012 15 4 2112 2118 doi:10.1167/iovs.11-8785 10.1167/iovs.11-8785 22427542

[B7] September AV Vorster AA Ramesar RS Greenberg LJ Mutation spectrum and founder chromosomes for the ABCA4 gene in South African patients with Stargardt disease Invest Ophthalmol Vis Sci 2004 15 6 1705 1711 10.1167/iovs.03-1167 15161829

[B8] Wiszniewski W Zaremba CM Yatsenko AN Jamrich M Wensel TG Lewis RA Lupski JR ABCA4 mutations causing mislocalization are found frequently in patients with severe retinal dystrophies Hum Mol Genet 2005 15 19 2769 2778 10.1093/hmg/ddi310 16103129

[B9] Marmor MF Fulton AB Holder GE Miyake Y Brigell M Bach M ISCEV Standard for full-field clinical electroretinography (2008 update) Doc Ophthalmol 2009 15 1 69 77 10.1007/s10633-008-9155-4 19030905

[B10] Nusinowitz S Nguyen L Radu R Kashani Z Farber D Danciger M Electroretinographic evidence for altered phototransduction gain and slowed recovery from photobleaches in albino mice with a MET450 variant in RPE65 Exp Eye Res 2003 15 5 627 638 10.1016/S0014-4835(03)00217-3 14550405

[B11] Nusinowitz S Sarraf D Retinal function in X-linked ocular albinism (OA1) Curr Eye Res 2008 15 9 789 803 10.1080/02713680802376353 18798082

[B12] Chen B Tosha C Gorin MB Nusinowitz S Analysis of autofluorescent retinal images and measurement of atrophic lesion growth in Stargardt disease Exp Eye Res 2010 15 2 143 152 10.1016/j.exer.2010.03.021 20398653

[B13] Clark MJ Chen R Lam HY Karczewski KJ Chen R Euskirchen G Butte AJ Snyder M Performance comparison of exome DNA sequencing technologies Nat Biotechnol 2011 15 10 908 914 10.1038/nbt.1975 21947028 PMC4127531

[B14] Li H Handsaker B Wysoker A Fennell T Ruan J Homer N Marth G Abecasis G Durbin R The sequence Alignment/Map format and SAMtools Bioinformatics 2009 15 16 2078 2079 10.1093/bioinformatics/btp352 19505943 PMC2723002

[B15] McKenna A Hanna M Banks E Sivachenko A Cibulskis K Kernytsky A Garimella K Altshuler D Gabriel S Daly M DePristo MA The genome analysis toolkit: a MapReduce framework for analyzing next-generation DNA sequencing data Genome Res 2010 15 9 1297 1303 10.1101/gr.107524.110 20644199 PMC2928508

[B16] NHLBI and NHGRI. Seattle Seq 2011 University of Washington

[B17] Grandinetti AA Portella E Arana J Iskorostenski NT Subretinal fibrosis in Stargardt’s disease: case report Arq Bras Oftalmol 2011 15 6 449 451 10.1590/S0004-27492011000600015 22331122

[B18] Cukras CA Wong WT Caruso R Cunningham D Zein W Sieving PA Centrifugal expansion of fundus autofluorescence patterns in Stargardt disease over time Arch Ophthalmol 2012 15 2 171 179 10.1001/archophthalmol.2011.332 21987580 PMC3768260

