# Supplementary material for: Whole exome sequencing detects homozygosity for ABCA4 p.Arg602Trp missense mutation in a pediatric patient with rapidly progressive retinal dystrophy
Source: BMC Med Genet. 2014 Jan 20;15:11. doi: 10.1186/1471-2350-15-11 (PMC3905103; doi:10.1186/1471-2350-15-11)
Supplement: Additional file 1 — Information for the 14 homozygous variants identified in subject meeting inclusion criteria: Quality Score ≥100, coverage depth ≥ 20, no overlap with segmental duplications (UCSC Genome Browser), Minor Allele Frequency <0.01 (dbSNP135). The putative causal variant in ABCA4 is highlighted in bold. No other variants are clinically relevant to the patient’s presentation. Abbreviations: Chr, chromosome; Ref, human genome reference nucleotide; Alt, alternate allele nucleotide; OMIM, Online Mendelian Inheritance in Man database; STGD, Stargardt Disease; OMD, Occult Macular Dystrophy; US1D, Usher Syndrome Type 1D. [file 1471-2350-15-11-S1.docx]

| Chr | Position | Ref | Alt | Depth | Gene | Transcript | Amino Acid Change | OMIM Disease(s) |
| --- | --- | --- | --- | --- | --- | --- | --- | --- |
| 1 | 86909555 | T | C | 124 | CLCA2 | NM_006536 | p.Met525Thr | n/a |
| **1** | **94528266** | **G** | **A** | **50** | **ABCA4** | **NM_000350** | **p.Arg602Trp** | **STGD, etc.** |
| 1 | 150972959 | A | T | 92 | FAM63A | NM_001040217 | p.Tyr95Asn | n/a |
| 1 | 158325745 | C | T | 97 | CD1E | NM_001042583 | p.Arg252Trp | n/a |
| 4 | 70361182 | T | C | 51 | UGT2B4 | NM_021139 | p.Asn133Ser | n/a |
| 4 | 71115159 | A | G | 49 | CSN3 | NM_005212 | p.Thr178Ala | n/a |
| 7 | 141971083 | A | C | 44 | LOC730441 | XM_002342801 | p.Asp122Glu | n/a |
| 10 | 73563128 | G | A | 41 | CDH23 | NM_022124 | p.Arg2608His | USH1D |
| 11 | 118425758 | G | C | 95 | C11orf60 | NM_020153 | p.Pro152Ala | n/a |
| 12 | 93251098 | C | G | 70 | EEA1 | NM_003566 | p.Glu107Gln | n/a |
| 13 | 22070236 | T | C | 50 | EFHA1 | NM_152726 | p.Ile333Val | n/a |
| 15 | 40005731 | T | A | 139 | FSIP1 | NM_152597 | p.Ile368Leu | n/a |
| 17 | 3324004 | C | T | 28 | OR3A3 | NM_012373 | p.Thr48Ile | n/a |
| 17 | 44144993 | C | G | 93 | KIAA1267 | NM_015443 | p.Arg525Pro | Koolen-De Vries syndrome |

Supplemental Table 1.

Information for the 14 homozygous variants identified in subject meeting inclusion criteria: Quality Score ≥100, coverage depth ≥ 20, no overlap with segmental duplications (UCSC Genome Browser), Minor Allele Frequency <0.01 (dbSNP135). The putative causal variant in ABCA4 is highlighted in bold. No other variants are clinically relevant to the patient’s presentation. Abbreviations: Chr, chromosome; Ref, human genome reference nucleotide; Alt, alternate allele nucleotide; OMIM, Online Mendelian Inheritance in Man database; STGD, Stargardt Disease; OMD, Occult Macular Dystrophy; USH1D, Usher Syndrome Type 1D.
